# Familial risk for depression is associated with reduced P300 and late positive potential to affective stimuli and prolonged cardiac deceleration to unpleasant stimuli

**DOI:** 10.1038/s41598-023-33534-z

**Published:** 2023-04-20

**Authors:** Tania Moretta, Simone Messerotti Benvenuti

**Affiliations:** 1 Department of General Psychology, University of Padua, Via Venezia, 8, 35131 Padua, Italy; 2 Padova Neuroscience Center (PNC), University of Padua, Padua, Italy; 3 Hospital Psychology Unit, Padua University Hospital, Padua, Italy

**Keywords:** Neuroscience, Physiology, Psychology, Biomarkers, Diseases, Risk factors

## Abstract

Despite evidence of abnormal affective processing as a key correlate of depression, specific attentional mechanisms underlying processing of emotions in familial risk for depression have yet to be investigated in a single study. To this end, the amplitude of the P300 and late positive potential (LPP) complex and cardiac deceleration were assessed during the passive viewing of affective pictures in 32 individuals who had family history of depression (without depressive symptoms) and in 30 controls (without depressive symptoms and family history of depression). Individuals with familial risk for depression revealed reduced P300-LPP amplitudes in response to pleasant and unpleasant stimuli relative to controls, and comparable P300-LPP amplitudes in response to pleasant and neutral stimuli. Controls, but not individuals with familial risk for depression, reported cardiac deceleration during the viewing of pleasant vs. neutral and unpleasant stimuli in the 0–3 s time window. Also, only individuals with familial risk for depression showed a prolonged cardiac deceleration in response to unpleasant vs. neutral stimuli. Overall, the present study provides new insights into the characterization of emotion-related attentional processes in familial risk for depression as potential vulnerability factors for the development of the disorder.

## Introduction

Depression is one of the most severe and common psychopathological conditions, affecting over 280 million people worldwide^1^. It is characterized by symptoms like sustained negative affect and anhedonia that negatively impact individuals’ life with impairments in occupational and psychosocial functioning, and an increased risk for suicide^2^.

Given its relevance, the comprehension of psychophysiological mechanisms involved in the risk of developing depression, such as the familial risk for depression, is needed to understand how depression is heritable, to early identify depression, and to develop novel and effective prevention programs^3^. Of note, to date, the most reliable risk factor for the development of major depressive disorder (MDD) is having a family history of the disorder^4,5^. Indeed, the estimated heritability of depression is about 37%^6^. However, despite advances in the knowledge of the psychobiology of MDD, no established mechanism can explain the risk of developing MDD^7,8^.

Previous studies investigating vulnerability factors for developing MDD have identified some personality traits^9–13^, blunted neural response to rewards^14–17^, reduced vagal control of the heart and higher levels of rumination^18,19^, and dysfunctional cognitive biases^20,21^. Specifically, cognitive processes have been shown to highly influence the development of MDD and MDD-related symptoms^22,23^. In cognitive models of depression, self-referential schemas negatively affect attention leading to a deficit in the cognitive resources available to process salient information. Individuals usually show a greater tendency to orient and sustain attention toward affective and salient than neutral cues^24,25^. According to cognitive models, individuals with depression are characterized by biased attention to mood-congruent stimuli, thereby processing negative information and filtering out positive information^22,26,27^, although some inconsistencies were observed^28,29^.

But then again, it was recently argued that avoidance of prospective rewards and thus directing attention away from positive information, would help to explain mechanisms underlying depressed states^30,31^. This view is in accordance with the hypothesis that attentional biases away from positive information are a part of the essential pathophysiology of depression, leading to reward devaluation^32,33^. Moreover, impairments of reward processing have been associated with dysregulated positive affect in depression^34^ and core depressive symptoms, such as anhedonia and social withdrawal^35^. Attentional biases away from positive information play also a key role in the matrix of the Positive Valence System within the Research Domain Criteria (RDoC) constructs, an initiative launched to identify the affective, cognitive, and neurophysiological features that characterize mental disorders^36^. Of note, it has been shown that an impaired approach-related motivation in the Positive Valence Systems characterizes unipolar depression^37^.

Importantly, the hypothesis of reduced processing of positive information as a key feature of depression has been extended, and blunted reactivity to all emotional stimuli (both pleasant and unpleasant) has been considered as one of the most important factors of depression^38–40^. These findings have been considered in formulating a theory called emotion context insensitivity (ECI)^41^. In the ECI, individuals with depression are thought to be characterized by dampening of reactivity to emotional stimuli in both positively- and negatively-valenced contexts^41^.

Following the view of the RDoC initiative of identifying constructs that reflect core mechanisms of psychopathology, the use of psychophysiological measures has been recommended as it plays a key role in the understanding of attentional processing of affective stimuli in individuals at risk of developing depression^42^. The use of event-related potentials (ERPs) has been largely acknowledged to study information processing in real-time during exposure to standardized emotional stimuli^43,44^. In particular, relative to neutral stimuli, high-arousing emotional stimuli typically elicit larger P300 and late positive potential (LPP) amplitudes in centro-parietal regions in the 300–700 ms time window. The P300 and LPP in response to affective information have been shown to reflect affective attentional allocation, stimuli representation, and evaluation^45^.

Of note, the P300 and LPP have been largely examined in depression as a possible correlate of dysfunctional affective processing of pleasant and/or unpleasant content. However, to the best of our knowledge, a larger LPP amplitude in response to unpleasant stimuli in individuals with depression has been found in a single study^46^. On the contrary, reduced LPP in response to threatening stimuli has been reported in both MDD^47,48^ and children of mothers with a history of depression^49^. Furthermore, reduced P300 and LPP amplitudes in response to pleasant stimuli have been largely documented in depression and risk for depression^17,32,50–52^ and to predict symptoms of depression^53^. Overall, findings on the P300 and LPP in response to affective information seem to suggest that both MDD and the risk of developing MDD are characterized by reduced affective attention to pleasant and unpleasant stimuli.

However, to date, there is only initial evidence suggesting that reduced P300 and LPP amplitudes in response to emotional stimuli may characterize familial risk for depression. Indeed, a study reported children with no lifetime depression but a maternal history of depression to be characterized by reduced LPP amplitude to pleasant and unpleasant relative to neutral faces^49^. This finding is in line with ECI theory^40^, suggesting that attenuated processing of affective stimuli may also represent an indicator of familial risk for depression. At the same time, Kayser et al.^54^ reported individuals with familial risk for depression, or a lifetime history of MDD to show reduced electrophysiological responsivity to unpleasant stimuli vs. neutral stimuli. These promising findings on emotional processing in familial risk for depression highlight the need for further studies in this context for a better understanding of the nature of the relation between attention to affective stimuli and risk for depression.

Depression has been also associated with impaired attentional processes during later stages of elaboration of affective information. Particularly, sustained attention to unpleasant information has been argued to be associated with negative affect^55^ and reflects a possible vulnerability factor for depression^56^. However, knowledge of later stages of attentional processes in individuals at risk for depression is still at an early stage, with the majority of studies using behavioral tasks such as the emotional Stroop task and the dot-probe task^57,58^. Using those tasks makes it difficult to discern between the processes of orienting and sustaining attention, failing to characterize later stages of attention processing^59,60^. Overall, this highlights the need of using different methodologies aimed at disentangling attentional processes among earlier and later stages of emotional processing to clarify the nature of attentional dysfunction in the risk for depression^57^.

A psychophysiological measure indicative of emotion-related attentional processes is cardiac deceleration. Specifically, heart rate changes during experienced emotional states have been shown to reflect specific (psycho)physiological processes in response to environmental demands. Indeed, during the viewing of high-arousing emotional stimuli, cardiac response is initially decelerative, indicating enhanced orienting and attention. Later in the process, cardiac response is accelerative, indicating motor preparation^24^. In passive tasks, greater cardiac deceleration has been considered as an index of the intention to note and detect external stimuli and readiness for effective actions^61^, whereas cardiac acceleration has been suggested to reflect a rejection of environmental stimuli^62^. Of note, while cortical activity mainly indicates recognition and memory of emotional stimuli^44^, heart rate reflects the transition from attentional processes to motor preparation^25,61^.

To the best of our knowledge, only two studies have investigated whether cardiac deceleration may reflect impaired emotional processing in dysphoria^15,63^. Of note, in both studies only individuals with dysphoria showed prolonged/sustained attention to unpleasant stimuli in the later stages of affective processing (3–6 s post-stimulus), suggesting sustained intake of unpleasant information^15,63^. The findings of these studies are consistent with the literature on abnormal affective processing in depression, which has been associated with alterations on later processes of autonomic reactivity to emotion^64^. Furthermore, findings on cardiac deceleration during emotional processing in dysphoria seem to suggest difficulties in disengaging attention from unpleasant information characterizing risk for depression.

In light of these considerations, specific attentional processes that underlie emotional processing in familial risk for depression have yet to be investigated in a single study. To address this gap, the P300-LPP complex and cardiac deceleration were assessed during a passive viewing task including pleasant, neutral, and unpleasant stimuli in young adults at high risk for the development of clinically significant depression, that is, individuals who had a family history of depression (but did not report current depressive symptoms). Based on the hypothesis of altered processing of pleasant or hedonic stimuli in depression and the ECI model on impaired processing of both pleasant and unpleasant information^32,39^, individuals with familial risk for depression were expected to be characterized by reduced P300-LPP amplitude in response to both pleasant and unpleasant stimuli, relative to controls (between-group hypothesis). Furthermore, differently from controls (no depressive symptoms and no family history of depression) who were expected to show greater P300-LPP amplitude in response to pleasant and unpleasant vs. neutral stimuli, it was hypothesized that the group with familial risk for depression would show comparable P300-LPP amplitude in response to affective (i.e., pleasant and unpleasant) and neutral stimuli (within-group hypothesis). Second, based on recent findings on cardiac deceleration in individuals with dysphoria^15,63^, individuals with familial risk for depression were also expected to be characterized by larger heart rate deceleration in response to unpleasant vs. neutral stimuli during later stages of affective processing, as compared to controls.

## Results

### Valence and arousal self-report ratings

A statistically significant main effect of Category was found for both valence and arousal ratings (valence: Χ_(2)_^2^ = 671.66, *p* < .001, ΔAIC = − 259; arousal: Χ_(2)_^2^ = 376.05, *p* < .001, ΔAIC = − 162). Unpleasant pictures were rated as significantly more unpleasant and arousing than pleasant and neutral pictures (all *p*s < .01). Furthermore, pleasant pictures were rated as significantly more pleasant and arousing than neutral pictures (*p*s < .001). No statistically significant effect of Group or Group × Category interaction emerged. The descriptive statistics of self-report measures are reported in Table 1.

**Table 1:** Valence and arousal self-report ratings for pleasant, neutral, and unpleasant stimuli in the two groups.

	Group with familial risk for depression (n = 32)			Group without familial risk for depression (n = 30)		
	Pleasant	Neutral	Unpleasant	Pleasant	Neutral	Unpleasant
Valence	6.6 ± 1.0	5.2 ± 0.7	2.9 ± 0.8	6.5 ± 0.9	5.4 ± 0.8	2.7 ± 0.9
Arousal	4.7 ± 1.9	2.2 ± 1.4	5.4 ± 1.6	5.1 ± 1.7	2.2 ± 1.3	5.7 ± 1.6

Data are mean ± standard deviation.

### P300-LPP complex peak

**Figure 1:**
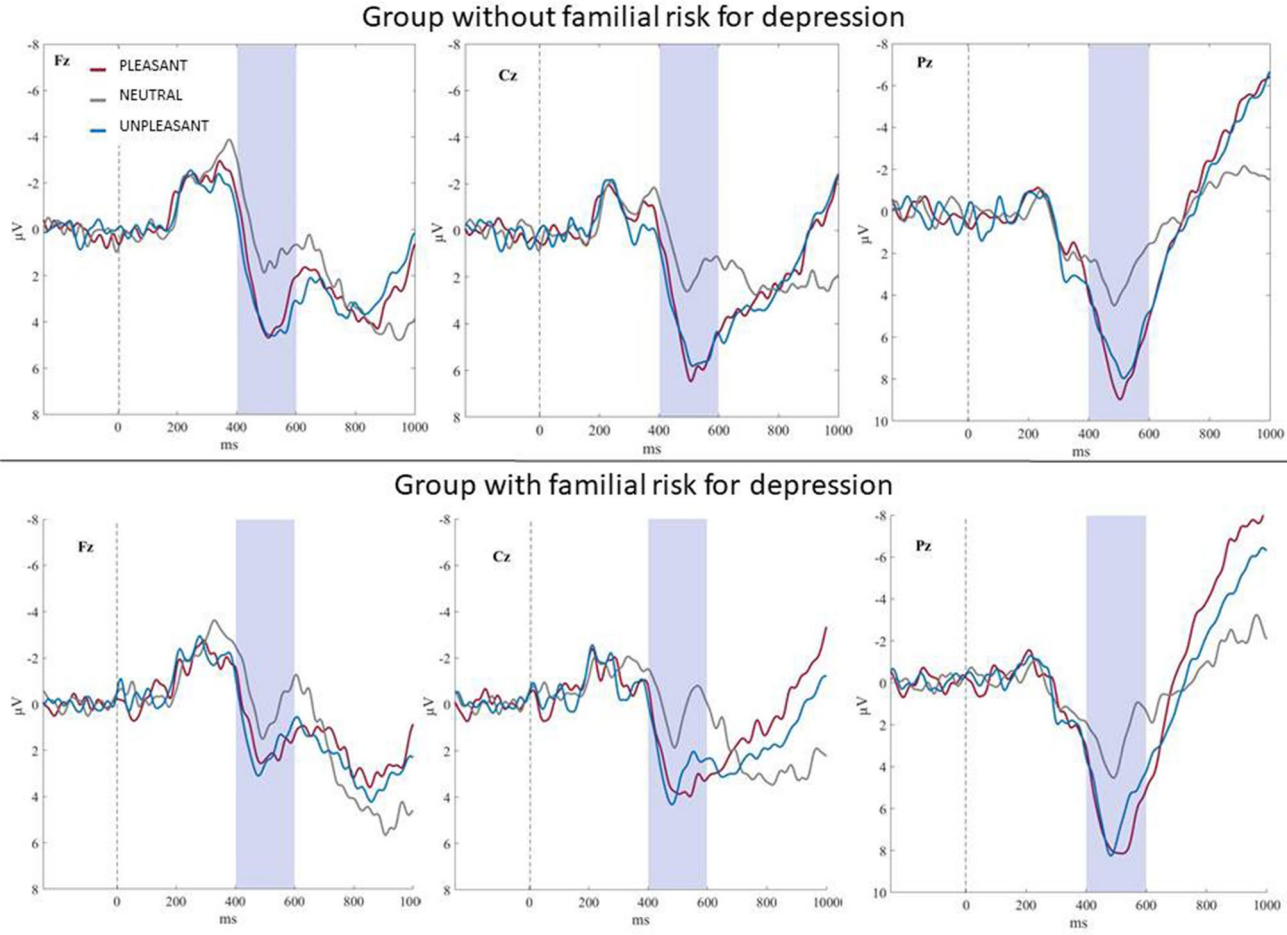
Grand-average ERP waveforms recorded at Fz, Cz, and Pz to pleasant, neutral, and unpleasant pictures in the group with and without familial risk for depression. The colored frame represents the 400–600 ms time window.

**Figure 2:**
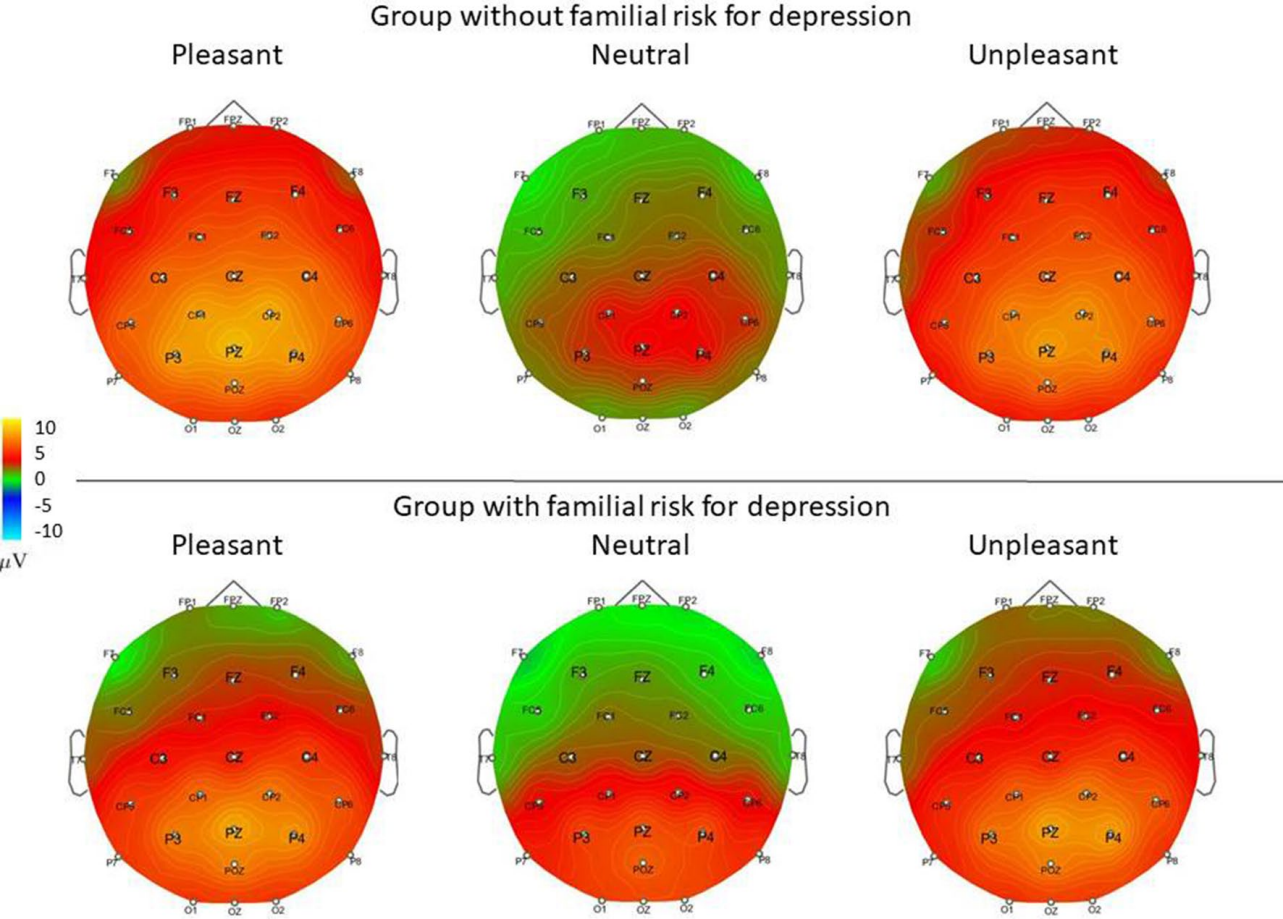
Scalp topography of pleasant, neutral, and unpleasant pictures in P300-LPP complex (400–600 ms) in the group with and without familial risk for depression.

Waveforms and scalp topography for each emotional category in the control group and the group with familial risk for depression are shown in Figs. 1 and 2.

**Figure 3:**
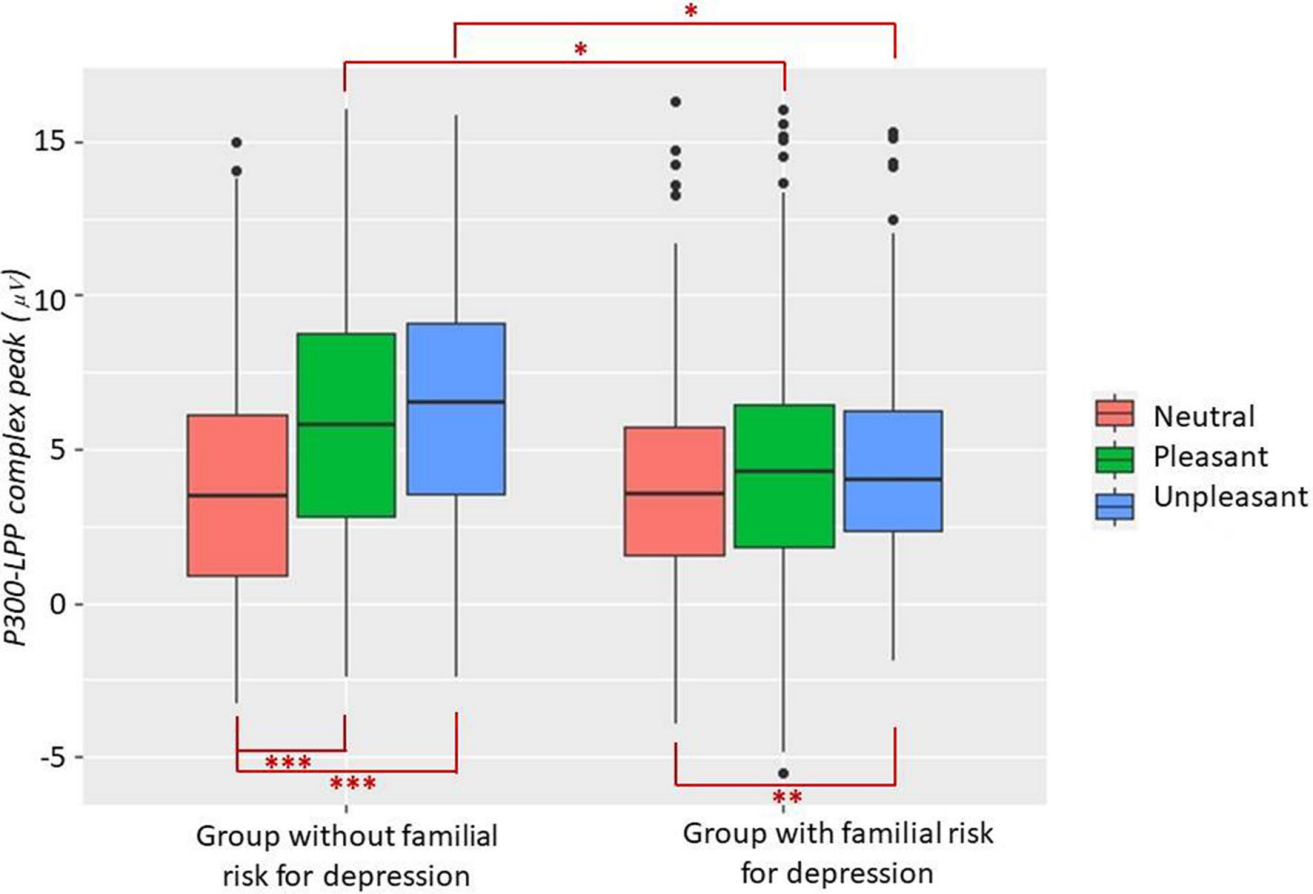
Boxplot of P300-LPP complex peaks (averaged over channels) for each emotional category in the group with and without familial risk for depression. On each box, the central mark is the median and the edges of the box are the 25th and 75th percentiles. *p < .05; **p < .01; ***p < .001.

The significant main effects of Category (F_2, 1560_ = 73.57, *p* < .001, ΔAIC = − 150) and Group (F_1, 60_ = 5.41, *p* = .02, ΔAIC = − 39) were further qualified by the significant Group × Category interaction (F_2, 1560_ = 24.25, *p* < .004, ΔAIC = − 24.8). Both groups showed larger P300-LPP amplitude in response to unpleasant than neutral stimuli (*p*s < .01). However, as shown in Fig. 3, whereas the control group showed larger positivity in response to pleasant than neutral pictures (*p* < .001), the P300-LPP amplitude showed no statistically significant difference between pleasant and neutral stimuli in the group with familial risk for depression (*p* = .14). Moreover, statistically significant between-group differences emerged in the P300-LPP amplitude in response to pleasant and unpleasant pictures. Relative to the control group, the group with familial risk for depression was characterized by lower positivity in response to both pleasant (*p* = .02) and unpleasant pictures (*p* = .01). In contrast, no statistically significant difference between the two groups emerged with respect to P300-LPP amplitude in response to neutral stimuli.

The significant main effect of Area (F_2, 1560_ = 388.51, *p* < .001, ΔAIC = − 607) showed larger P300-LPP amplitude in the parietal than the central and the frontal areas (*p*s < .001), and lower positivity in the frontal than the central area (*p* < .001).

### P300-LPP complex latency

The statistically significant effects of Category (F_2, 1560_ = 41.31, *p* < .001, ΔAIC = − 209), Area (F_2, 1560_ = 13.97, *p* < .001, ΔAIC = − 256), and Group × Area interaction (F_2, 1560_ = 4.49, *p* = .01, ΔAIC = − 309) were further qualified by the significant Category × Group × Area interaction (F_4, 1560_ = 3.53, *p* = .01, ΔAIC = − 329). P300-LPP complex latency was longer for pleasant and unpleasant than for neutral pictures in the central area in both groups (*p*s < .03), and in the parietal area for the group with familial risk for depression only (*p*s < .03). Moreover, only individuals with familial risk for depression showed longer P300-LPP complex latency in the parietal area than in the frontal and central areas for pleasant pictures (*p*s < .001). No other statistically significant differences emerged.

### Heart rate deceleration

**Figure 4:**
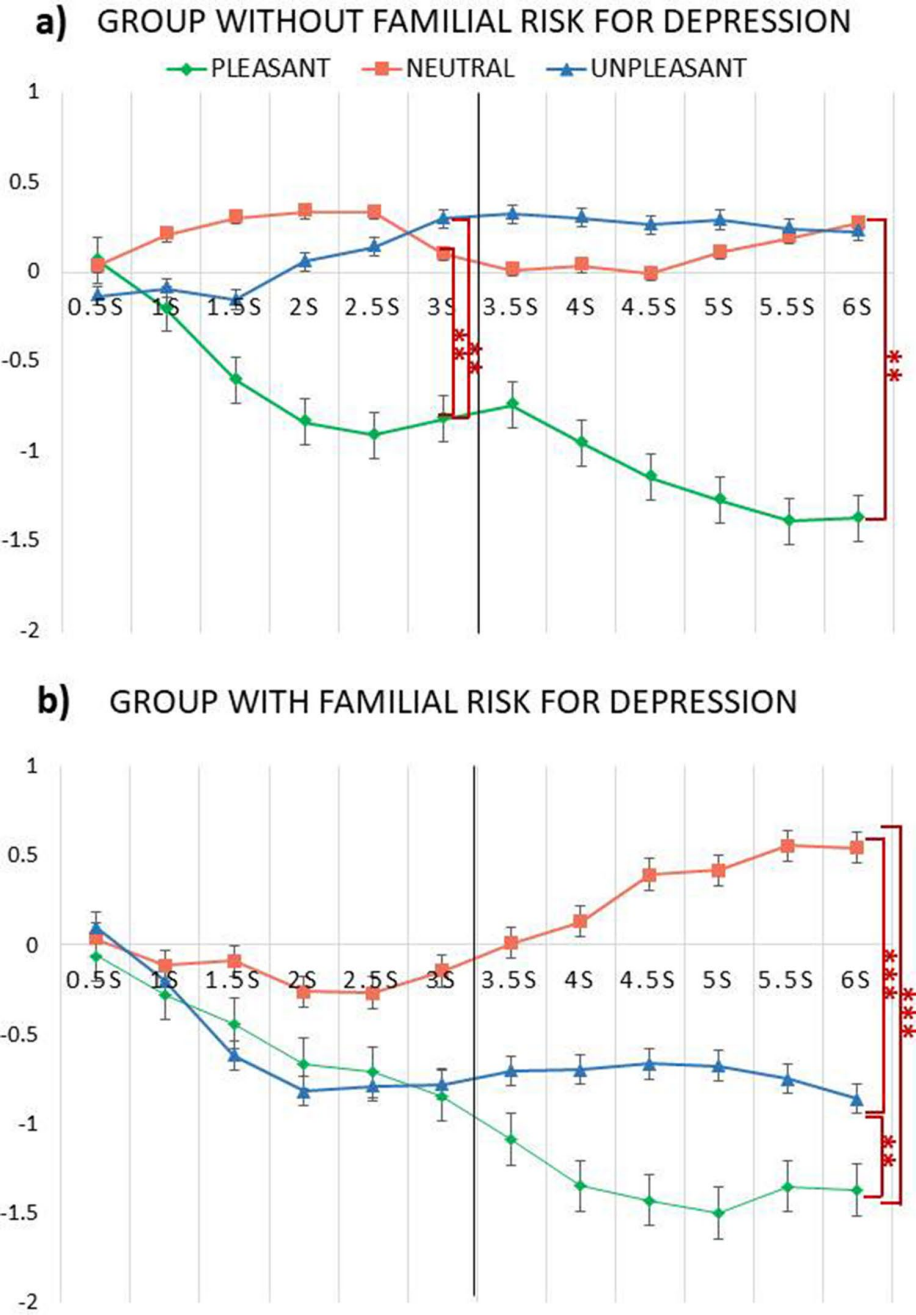
Averaged heart rate change during the viewing of pleasant, neutral, and unpleasant pictures in the group without (**a**) and with (**b**) familial risk for depression. Units are beats per minute (bpm) changes from 2 s baseline. Error bars represent ± standard error of the mean (SEM). **p < .01; ***p < .001.

The LMM showed a statistically significant effect of Category, F_(2, 1940)_ = 101.13, *p* < .001, ΔAIC = − 241, Time, F_(1, 10)_ = 7.26, *p* = .02, ΔAIC = − 39.1, Category × Time interaction, F_(1, 1940)_ = 22.28, *p* < .001, ΔAIC = − 38.8, and Category × Group interaction, F_(2, 1940)_ = 15.14, *p* < .001, ΔAIC = − 26.4, which were further qualified by a significant Group × Category × Time interaction, F_(2, 1940)_ = 7.38, *p* < .001, ΔAIC = − 59.8. As shown in Fig. 4a,b, in the 0–3 s time window, heart rate deceleration was larger during the viewing of pleasant than neutral and unpleasant pictures in the control group, whereas no statistically significant differences between the three emotional categories emerged with respect to heart rate deceleration in the group with familial risk for depression. In both groups, heart rate deceleration was larger during the viewing of pleasant pictures in the 3–6 s than the 0–3 s time window in both groups (all *p*s < .01).

Of note, in the group with familial risk for depression, but not in the control group, heart rate deceleration was larger during the viewing of unpleasant than neutral pictures in the 3–6 s time window (*p* < .001; Fig. 4). Related to this, statistically significant differences between the two groups were also found with the group with familial risk for depression showing larger heart rate deceleration during the viewing of unpleasant pictures in the 3–6 s time window as compared with the control group (*p* = .02).

## Discussion

To the best of our knowledge, the present study represents the first attempt to investigate emotional processing in individuals with familial risk for depression by using both central and peripheral psychophysiological measures. Specifically, the P300-LPP complex and heart rate deceleration were investigated in individuals with vs. without familial risk for depression during passive viewing of affective pictures. Based on previous findings on altered processing of positive information in depression and based on ECI model, it was hypothesized that as compared to controls, individuals with familial risk for depression would show reduced P300-LPP amplitudes in response to pleasant and unpleasant stimuli relative to controls and comparable P300-LPP amplitudes in response to pleasant, unpleasant, and neutral stimuli (e.g.,^32,39^). Moreover, individuals with familial risk for depression were expected to show larger heart rate deceleration in response to unpleasant than neutral stimuli relative to controls^15,63^.

In line with our hypothesis, individuals with familial risk for depression showed reduced P300-LPP amplitudes in response to pleasant and unpleasant stimuli relative to controls, and comparable P300-LPP amplitudes only in response to pleasant and neutral stimuli. Of note, P300-LPP amplitudes in response to pleasant stimuli were smaller in the group with familial risk for depression than in the control group. This result is consistent with previous findings on clinical depression, indicating that a reduction in sustained cortical positivity to rewarding information may be a correlate of abnormal affective attentional allocation to pleasant and hedonic stimuli^65^. Furthermore, this finding suggests that reduced affective attention towards positive/rewarding stimuli is not only a feature of individuals with clinical symptoms of depression^15,38,62,65^ or subclinical depression^15^, but also of those with familial risk for depression without current depressive symptoms. Regarding the RDoC framework, these findings support the proposed hypothesis of a hypoactivation of the Positive Valence System as a key mechanism underlying depressed mood^37,66^.

It is worth noting that other than P300-LPP amplitudes in response to pleasant stimuli, late cortical positivity in response to unpleasant stimuli was also reduced in individuals at familial risk for depression relative to controls. Moreover, individuals with familial risk for depression showed longer P300-LPP complex latency in response to emotional (pleasant and unpleasant) vs. neutral stimuli suggesting difficulties in emotional processing. Reduced processing capacity of both pleasant and unpleasant contents can be hypothesized to be a key feature characterizing particularly individuals with familial risk for depression. Of note, this finding supports the predictions of the ECI model on processing of both pleasant and unpleasant information^38,40^. However, individuals at familial risk for depression showed larger P300-LPP amplitude in response to unpleasant than neutral stimuli. Larger confirmatory studies comparing emotional processing in individuals with and without both symptoms of depression and familial risk for depression are needed to further investigate whether reduced affective attention to unpleasant information is a peculiar feature characterizing familial risk for depression.

With respect to cardiac deceleration, the pattern of heart rate changes in individuals with familial risk for depression vs. controls differed as a function of emotional condition. Only controls reported cardiac deceleration during the viewing of pleasant vs. neutral and unpleasant stimuli in the 0–3 s time window. This finding seems to suggest delayed orienting of attention towards pleasant contents characterizing familial risk for depression. Moreover, this result is in accordance with studies reporting compromised affective attention allocation to pleasant information during earlier stages of processing in individuals at risk for depression^15^. Importantly, the fact that both groups showed the typical heart rate deceleration during the viewing of pleasant vs. neutral stimuli in the 3–6 s time window^67^ suggests that processing of pleasant content is delayed in individuals with familial risk for depression, with defective attention allocation/orienting during earlier stages and preserved sustained attention during later stages^15^.

Of note, only individuals with familial risk for depression showed a sustained cardiac deceleration in response to unpleasant than neutral stimuli (3–6 s time window). This finding is consistent with the hypothesis that risk for depression is characterized by impaired disengagement from unpleasant information in later stages of attentional processes^15,63^. Deficits in inhibition and modulation of processing of unpleasant information have been related to maladaptive emotion regulation and difficulties in recovering from negative affect^23,34^. These deficits have been linked to maladaptive repetitive self-focused thoughts leading to rumination^68^ which, in turn, has been recently described as an early indicator of vulnerability to depression^18^. The result on later stages of emotional processing seems to be in contrast with a prediction of the ECI model on reduced reactivity to unpleasant stimuli. However, sustained cardiac deceleration has been associated with both sensory intake of unpleasant stimuli and inhibition of readiness for actions, thus reflecting the underactivation of the Negative Valence motivational system^41^. Future studies should further assess differences in affective attention vs. motivation to action in individuals at risk of developing depression and their relationships with emotion regulation and rumination as possible vulnerability factors in this population.

It should be noted that no cardiac deceleration in response to unpleasant pictures in the earlier stages of processing (0–3 s) was observed in both groups^15^. This finding is in contrast with those reporting marked deceleration at the initial stages of picture processing in response to unpleasant content^67^. It has been suggested that absence of cardiac deceleration in response to unpleasant stimuli would depend on an age-related bias since younger adults perceive unpleasant pictures as less negative^69^. Thus, considering that in the present study participants were young adults, unpleasant pictures may have not been perceived by controls negative and aversive enough to trigger enhanced orienting.

With respect to subjective measures, in the present study, self-report ratings of arousal and valence did not differ between the two groups. This finding is consistent with those of previous studies on subclinical^15,16,70,71^ and clinical depression^72^. Taken together these results suggest that group differences in the P300-LPP amplitudes and heart rate deceleration cannot depend on valence and arousal subjective ratings. Moreover, these findings would indicate that ERPs and cardiac deceleration are more sensitive measures than subjective ratings to assess vulnerability to depression. Specifically, as compared to subjective ratings of valence and arousal, these psychophysiological measures are able to assess unaware emotional processing that may reflect the abnormal patterns of affective attentional processes in individuals at risk to develop depression.

From a clinical perspective, the present study suggests that reduced affective attentional allocation towards pleasant and unpleasant content and difficulties in disengaging attention from unpleasant information may be a correlate of familial risk for depression. Therefore, findings on abnormal affective disposition and attentional processes in individuals with familial risk for depression may inform preventive programs. Accordingly, interventions specifically aimed at increasing motivation for action, such as the behavioral activation treatment^73^, may be used to prevent depressive symptoms in at-risk individuals. Attention bias modification procedures could be also adopted to orient attention towards pleasant information and away from unpleasant stimuli^74^.

The current findings should be interpreted in light of some methodological limitations. First, the present study has been conducted as a first hypothesis testing and should be considered to design larger confirmatory studies. Second, although the groups did not differ in terms of sex distribution, the participants in the current study were predominately female. The results presented may be more generalizable to females than males. Third, only a longitudinal study will be able to establish whether the current ERPs and cardiac deceleration results reflect an abnormal psychophysiological pattern characterizing familial risk for depression.

Overall, the results of the current study showed that familial risk for depression is characterized by a neural profile of attenuated affective attention to positive information and by a heart rate profile of delayed orienting towards pleasant contents and sustained attention toward unpleasant information. These patterns of emotional processing of affective stimuli may be specific for the familial risk and may represent an early indicator to identify those individuals at risk of depression.

## Methods

### Participants

A total of 62 students of the University of Padua, Italy, voluntarily took part in the study. Seventeen of the 62 participants had also participated in a previous study on emotional processing in dysphoria^15^. The enrolled sample was medically healthy and free from psychotropic medication (e.g., antidepressant medication) and/or drugs of abuse, as assessed with an ad-hoc interview.

Given that the present study is the first to investigate specific attentional processes that underlie emotional processing in familial risk for depression, there was no related effect size to choose from for formal power analysis. The present study has been conducted as a first hypothesis testing and should be used to design larger confirmatory studies. At the beginning, we aimed to recruit about 60 students. In practice, we were able to collect data from 62 participants by the end of the academic year.

In order to identify participants with familial risk for depression without depressive symptoms, the Family History Screen (FHS)^75^ was administered to assess the presence of current or past MDD and/or other psychopathological conditions in first-degree relatives. Moreover, module A of the Structured Clinical Interview for DSM-5-Clinical Version (SCID 5-CV)^76,77^ was also administered to assess current and past depressive symptoms. Furthermore, the Beck Depression Inventory-II (BDI-II)^78,79^ was employed to assess depressive symptoms’ severity. Based on the psychological assessment, 32 participants who scored equal to or lower than 12 on the BDI-II, without meeting the diagnostic criteria for a major depression episode, persistent depressive disorder, or bipolar disorder and had at least one first-degree relative with a history of MDD (i.e., parent and/or sibling) were assigned to the group with familial risk for depression (demographic and clinical characteristics are reported in Table 2). In this group, among biological relatives with symptoms of MDD, 11.2% of participants indicated their father, 48.1% their mother, 25.9% their sibling, 14.81% reported more than two relatives with symptoms of MDD; moreover, 63.6% of participants reported that one of their biological relatives experienced both symptoms of MDD included in the FHS.

**Table 2:** Demographic and clinical characteristics in the two groups.

	Group with familial risk for depression	Group without familial risk for depression	Test-statistic	*p* value
N (female %)	32 (72%)	30 (70%)	− 0.16^Glm, z test^	.87
Age (year)	21.9 ± 3.3	21.0 ± 3.2	1.06^t test^	.29
Education (year)	16.1 ± 2.7	15.2 ± 2.4	1.56^t test^	.12
Sleep hours per day	7.1 ± 0.8	7.3 ± 1.0	− 0.79^t test^	.43
Cigarette consumption per day	0.9 ± 2.2	0.9 ± 1.2	0.04^t test^	.97
MDD DSM-5, n (%)				
Current	0 (0%)	0 (0%)	–	–
Past	18 (56%)	12 (40%)	1.27^Glm, z test^	.20
BDI-II	5.4 ± 3.5	4.8 ± 3.1	0.58^t test^	.57

Continuous data are mean ± standard deviation; *Glm* = generalized linear model with binomial error distribution, *MDD* major depressive disorder, *BDI-II* Beck Depression Inventory-II.

Thirty participants who scored equal to or lower than 12 on the BDI-II, without meeting the diagnostic criteria for a major depression episode, persistent depressive disorder, or bipolar disorder and had no first-degree relative with a history of MDD were assigned to the control group (demographic and clinical characteristics are reported in Table 2). As shown in Table 2, the two groups did not differ in terms of sex distribution, age, years of education, sleep hours, cigarette consumption per day, current and past episodes of depression, and BDI-II scores.

Participants were compensated 13 € for their participation. All participants understood and signed informed consent forms. The study was conducted in compliance with the World Medical Association Declaration of Helsinki on research on human subjects and was approved by the Ethical Committee of Psychological Research, Area 17, University of Padova (prot. no. 3712).

The data that support the findings of this study are available on request from the corresponding author, TM. The data are not publicly available due to information that could compromise the privacy of research participants.

### Psychological measures

The Italian version of the FHS^75^ was administered as a reliable structured interview to assess the presence of family psychiatric conditions in biological relatives (i.e., biological parents, siblings). The FHS assesses information on 15-lifetime psychiatric disorders and suicide attempts. In particular, in the beginning, participants were asked to endorse general questions about psychopathological features, treatment, and impairment of their biological relatives, followed by more specific questions about psychopathological features during the entire lifetime of all family members. In the present study, an affirmative answer to the question “Did one of your parents or sibling ever have a period of feeling sad, blue, or depressed for most of the time for at least two weeks? (Please answer by reporting the member of your family who experienced these feelings without including time of physical illness or mourning after a death)” and/or to the question “Did one of your parents or sibling ever have a period (at least two weeks) of feeling quite tired, having less energy, or not caring about their usual activities? (Please answer by reporting the member of your family who experienced these feelings without including time of physical illness or mourning after a death)” was considered as indicative of a first-degree relative with a history of MDD. The FHS showed high sensitivity^80^ and validity for major depression, anxiety disorders, substance use disorder, and suicide attempts^75^.

The Italian version of the mood episode module (module A) of the SCID-5-CV^77^ was administered as a reliable tool to exclude individuals with major depression, persistent depressive disorder, or bipolar disorder. The module was administered by a trained psychologist who had previous experience with administering structured clinical interviews.

The Italian version of the BDI-II^79^ was administered as a reliable self-report questionnaire assessing the severity of depressive symptoms in the past two weeks. The BDI-II includes 21 items, each with a four-point Likert scale and scores ranging from 0 to 63, with higher scores indicating greater depressive symptoms. In the Italian version, a score of 12 has been reported as the optimal cut-off score to discriminate between individuals with and without depressive symptoms^79^. For this study, the Cronbach's Alpha was α = .91 indicating high internal consistency.

### Experimental task and procedure

The task used in this study is the same one used previously in our laboratory^15,81^. Twenty-four pleasant (i.e., erotic couples, sports), 24 neutral (i.e., neutral faces, household objects), and 24 unpleasant (i.e., attacking humans and animals) color pictures (600 × 800 pixels) were presented to participants. Highly arousing pleasant and unpleasant pictures selected from the International Affective Picture System^82^ were chosen to induce remarkable psychophysiological changes^24,83^. Pleasant and unpleasant pictures were matched for normative arousal ratings and were significantly higher than neutral pictures (*p* < .001).

Pictures were presented for 6000 ms each in a semi-randomized sequence (i.e., no more than one stimulus in the same emotional condition had to be shown consecutively). Each picture was preceded by a 3000 ms gray interval with a white fixation cross placed centrally on the screen. Participants had to look at the central fixation cross. A variable intertrial interval (ITI) of 6000–8000 ms, including a white fixation cross identical to the 3-s baseline, followed each picture.

Participants had to avoid alcohol consumption the day before the appointment and caffeine and nicotine on the same day of the appointment. On the day of the experimental session, after reading and signing the informed consent, participants were administered the ad-hoc anamnestic interview, the module A of the SCID-5-CV, the FHS, and the BDI-II. Then, participants were seated on a comfortable chair in a dimly lit, sound-attenuated room. After electrodes attachment and a 3-min resting-state period, six practice trials including two pleasant, two neutral, and two unpleasant pictures were provided. Then, the emotional passive viewing task was presented. At the end of the task, 36 pictures (12 for each emotional category) were shown again, and ratings of emotional valence and arousal were obtained via a computerized version of the 9-point Valence and Arousal scales of the Self-Assessment Manikin (SAM)^84^. The procedure lasted about 90 min.

### Apparatus and physiological recording

Apparatus and physiological recording is similar to those described in previous studies conducted in our laboratory^15,81^. Physiological measures were recorded in a standardized fashion using a computer running eego™ software and an eego amplifier (ANT Neuro, Enschede, Netherlands). The electroencephalogram (EEG) was recorded using an elastic cap with 32 tin electrodes arranged according to the 10–20 System (Fp1, Fpz, Fp2, F7, F3, Fz, F4, F8, FC5, FC1, FC2, FC6, T7, C3, Cz, C4, T8, CP5, CP1, CP2, CP6, P7, P3, Pz, P4, P8, POz, O1, Oz, O2, and M1 and M2 [mastoids]), referenced online to CPz. Vertical and horizontal electrooculograms (EOGs) were recorded using a bipolar montage. Electrodes were placed at the supra- and suborbit of the right eye and the external canthi of the eyes. Electrode impedance was kept below 10 kΩ. The EEG and EOG signals were amplified, bandpass filtered (0.3–40 Hz), and digitized at 1000 Hz.

The electrocardiogram (ECG) was recorded using Ag/AgCl surface electrodes that were positioned on the participant's chest in a modified lead II configuration. The ECG signal was amplified, band-pass filtered (0.3–100 Hz), and stored on a Core 2 Quad computer. The ECG was sampled at 1000 Hz and the electrode impedance was kept below 5 kΩ.

### Data reduction and analysis

In order to decrease computation time, the EEG data was downsampled to 500 Hz. Moreover, data was re-referenced offline to a linked mastoids montage by EEGLAB toolbox^85^. Further processing was carried out in Brainstorm^86^. The EEG was filtered offline with a band-pass filter of 0.3–30 Hz and manually corrected for blink artifacts via independent component analysis. The EEG was then segmented into 6000 epochs, from 3000 ms before- to 3000 ms after the stimulus onset. Each epoch was baseline-corrected by subtracting the mean pre-stimulus voltage between − 250 ms and − 50 ms. Then, EEG epochs were visually inspected for eye movements and other artifacts, and each portion of data containing residual artifacts exceeding ± 70 μV (peak-to-peak) was excluded. The artifact rejection led to an average ± SD acceptance of 21.9 ± 2.2 pleasant trials, 21.4 ± 2.4 neutral trials, and 21.7 ± 2.8 unpleasant trials in the group with familial risk for depression, and 21.4 ± 2.4 pleasant trials, 22.0 ± 2.5 neutral trials, and 21.8 ± 2.4 unpleasant trials in the control group. No statistically significant differences between groups and among emotional conditions in the average acceptance rate were found (all *p*s > .20). In the present study, previous findings were used to guide the selection of both time window and electrodes as it is considered as an adequate approach in well-established study design like passive viewing task with affective stimuli^43,87^. According to the literature^43,87–89^ and visual inspection of the grand-average ERPs waveforms, peaks were calculated in the 400–600 ms time window for the P300-LPP complex at F3, Fz, F4, C3, Cz, C4, P3, Pz, P4.

The ECG was analyzed offline 2000 ms before picture onset (baseline) and during 6000 ms of picture presentation using the Biopac Acqknowledge 5.0 software (Biopac Systems Inc., USA). A digital trigger detecting R-waves was applied to the ECG signal to obtain RR intervals, corresponding to the inverse of heart rate. Data were then visually inspected and six participants in the group with familial risk for depression were excluded due to extended artifacts in the ECG signal. Data were reduced offline in half-second bins according to the harmonic mean criterion (Graham, 1980), using the Matlab software KARDIA (MathWorks Inc., Natick, MA, USA). Heart rate deceleration was obtained by subtracting each heart rate value from that measured during the baseline period.

### Statistical analysis

Valence and arousal self-report ratings were analyzed by separate linear mixed-effect models (LMMs) individual random intercept and Category (i.e., pleasant, neutral, unpleasant) and Group (i.e., individuals with and without familial risk for depression) as fixed factors.

LMMs with individual random intercept were conducted on both mean P300-LPP complex peaks and P300-LPP complex latecies with Category, Group, Area (frontal [F3, Fz, F4], central [C3, Cz, C4], and parietal [P3, Pz, P4]), Laterality (left [F3, C3, P3], midline [Fz, Cz, Pz], right [F4, C4, P4]) and their interaction as fixed factors.

An LMM with individual and half-second bins as random intercepts was also conducted on heart rate deceleration data, with Category, Group, and Time (0–3 s, 3–6 s) as fixed factors.

In all LMMs the strength of parameters evidence within the models was estimated as the difference in the Akaike information criterion (AIC) between the model with and the model without the parameter (ΔAIC)^90^. Denominator degrees of freedom were estimated by Satterthwaite and Kenward-Roger methods^91^. Bonferroni HSD post-hoc tests were employed to further examine significant effects (*p* < .05).

## Data Availability

The datasets analyzed during the current study are not publicly available due to ethical concerns but are available from the corresponding author on reasonable requests.
